# GC–MS and LC-TOF–MS profiles, toxicity, and macrophage-dependent in vitro anti-osteoporosis activity of *Prunus africana* (Hook f.) Kalkman Bark

**DOI:** 10.1038/s41598-022-10629-7

**Published:** 2022-04-29

**Authors:** Richard Komakech, Ki-Shuk Shim, Nam-Hui Yim, Jun Ho Song, Sungyu Yang, Goya Choi, Jun Lee, Yong-goo Kim, Francis Omujal, Denis Okello, Moses Solomon Agwaya, Grace Nambatya Kyeyune, Hyemin Kan, Kyu-Seok Hwang, Motlalepula Gilbert Matsabisa, Youngmin Kang

**Affiliations:** 1 Herbal Medicine Resources Research Center, Korea Institute of Oriental Medicine (KIOM), 111 Geonjae-ro, Naju-si, Jeollanam-do 58245 Republic of Korea; 2 University of Science and Technology (UST), Korean Convergence Medicine Major, KIOM campus, 1672 Yuseongdae-ro, Yuseong-gu, Daejeon, 34054 Republic of Korea; 3 Korea Institute of Oriental Medicine (KIOM), 1672 Yuseongdae-ro, Yuseong-gu, Daejeon, 34054 Republic of Korea; 4 Korean Medicine Application Center, Korea Institute of Oriental Medicine, 70 Cheomdan-ro, Dong-gu, Daegu, 41062 Republic of Korea; 5 Natural Chemotherapeutics Research Institute (NCRI), Ministry of Health, P.O. Box 4864, Kampala, Uganda; 6 Bio and Drug Discovery Division, Korea Research Institute of Chemical Technology, Daejeon, Republic of Korea; 7 IKS Research Group, Department of Pharmacology, Faculty of Health Sciences, University of the Free State, Bloemfontein, 9301 Free State South Africa

**Keywords:** Biochemistry, Drug discovery, Plant sciences, Diseases

## Abstract

Osteoporosis affects millions of people worldwide. As such, this study assessed the macrophage-dependent in vitro anti-osteoporosis, phytochemical profile and hepatotoxicity effects in zebrafish larvae of the stem bark extracts of *P. africana*. Mouse bone marrow macrophages (BMM) cells were plated in 96-well plates and treated with *P. africana* methanolic bark extracts at concentrations of 0, 6.25, 12.5, 25, and 50 µg/ml for 24 h. The osteoclast tartrate-resistant acid phosphatase (TRAP) activity and cell viability were measured. Lipopolysaccharides (LPS) induced Nitrite (NO) and interleukin-6 (IL-6) production inhibitory effects of *P. africana* bark extracts (Methanolic, 150 µg/ml) and β-sitosterol (100 µM) were conducted using RAW 264.7 cells. Additionally, inhibition of IL-1β secretion and TRAP activity were determined for chlorogenic acid, catechin, naringenin and β-sitosterol. For toxicity study, zebrafish larvae were exposed to different concentrations of 25, 50, 100, and 200 µg/ml *P. africana* methanolic, ethanolic and water bark extracts. Dimethyl sulfoxide (0.05%) was used as a negative control and tamoxifen (5 µM) and dexamethasone (40 µM or 80 µM) were positive controls. The methanolic *P. africana* extracts significantly inhibited (*p* < 0.001) TRAP activity at all concentrations and at 12.5 and 25 µg/ml, the extract exhibited significant (*p* < 0.05) BMM cell viability. NO production was significantly inhibited (all p < 0.0001) by the sample. IL-6 secretion was significantly inhibited by *P. africana* methanolic extract (p < 0.0001) and β-sitosterol (p < 0.0001) and further, chlorogenic acid and naringenin remarkably inhibited IL-1β production. The *P. africana* methanolic extract significantly inhibited RANKL-induced TRAP activity. The phytochemical study of *P. africana* stem bark revealed a number of chemical compounds with anti-osteoporosis activity. There was no observed hepatocyte apoptosis in the liver of zebrafish larvae. In conclusion, the stem bark of *P. africana* is non-toxic to the liver and its inhibition of TRAP activity makes it an important source for future anti-osteoporosis drug development.

## Introduction

Osteoporosis is a silent but one of the major global health problems characterized by deterioration of bone microarchitecture and low bone mass^1,2^. It is one of the leading causes of morbidity in older people above 40 years^3^. This condition occurs when the rate of bone resorption is higher than the rate of bone formation and consequently presents a greater risk of fractures for the persons suffering from it. Factors such as aging, sex steroid deficiency, and as well as menopause in women have been associated with a higher risk of osteoporosis^4^. Activated macrophages have also been implicated in the pathogenesis of osteoporosis by stimulating the development of osteoclastogenesis-associated bone loss^5^. Cytokines such as tumor necrosis factor alpha (TNFα), interleukin-1 (IL-1β) and interleukin-6 (IL-6) are pro-inflammatory and play central role in inflammation of which IL-6 is the most important in chronic inflammatory and autoimmune diseases, cytokine storm and cancer^6,7^. IL-6 is fundamental in a number of processes including bone metabolism, inflammation, hematopoiesis^7^. IL-6 is also implicated in mediation of IL-1 effects, a potent bone resorption stimulator^8^. Although a number of conventional methods have been employed in the treatment of osteoporosis including bisphophates and estrogen hormonal therapy, adverse effects associated with these therapies that are reported to limit their use include; gastrointestinal tract disturbances and burning sensation^9^. Exploration of other avenues including the use of natural products in the treatment of osteoporosis have been suggested to offer a better alternative with lesser adverse side effects^9^. Herbal medicines have been used over the years to prevent and treat osteoporosis condition^2^. The anti-osteoporosis of herbal products has been attributed to the secondary metabolites including alkaloids, terpenes, steroids, and phenolic compounds^10,11^ and these have led to the development of a number of drugs over the years with great therapeutic activities^12^. *Prunus africana* (Hook f.) Kalkman (Family Rosaceae), commonly called African cherry is an evergreen plant endemic in sub-Saharan Africa^13^, and contains a number of secondary metabolites like terpenes, alkaloids, phenolic compounds, and sterols in its stem bark^14^. For centuries, *P. africana* has been used in Africa to treat myriad of diseases including prostate cancer, hyperplasia, diabetes, malaria, and inflammatory conditions^15^. Despite its immense medicinal uses and with a wide array of secondary metabolites, *P. africana* has not been investigated for anti-osteoporosis activity yet it is used by the persons with prostate cancer who are vulnerable to suffer from osteoporosis^16,17^. Hence, this study evaluated the phytochemistry *P. africana* bark extract and it’s in *vitro* anti-osteoporosis activity based on osteoclast tartrate-resistant acid phosphatase (TRAP) as a cytochemical marker of osteoclasts*.* However, due to the toxicity associated with some of the herbal medicines such as liver damage^18^ and owing to association of liver health and osteoporosis^19^, this study also evaluated the hepatotoxicity of *P. africana* bark extracts in zebrafish (*Danio rerio*) larvae.

## Materials and methods

### Plant material and preparation of extract

**Figure 1:**
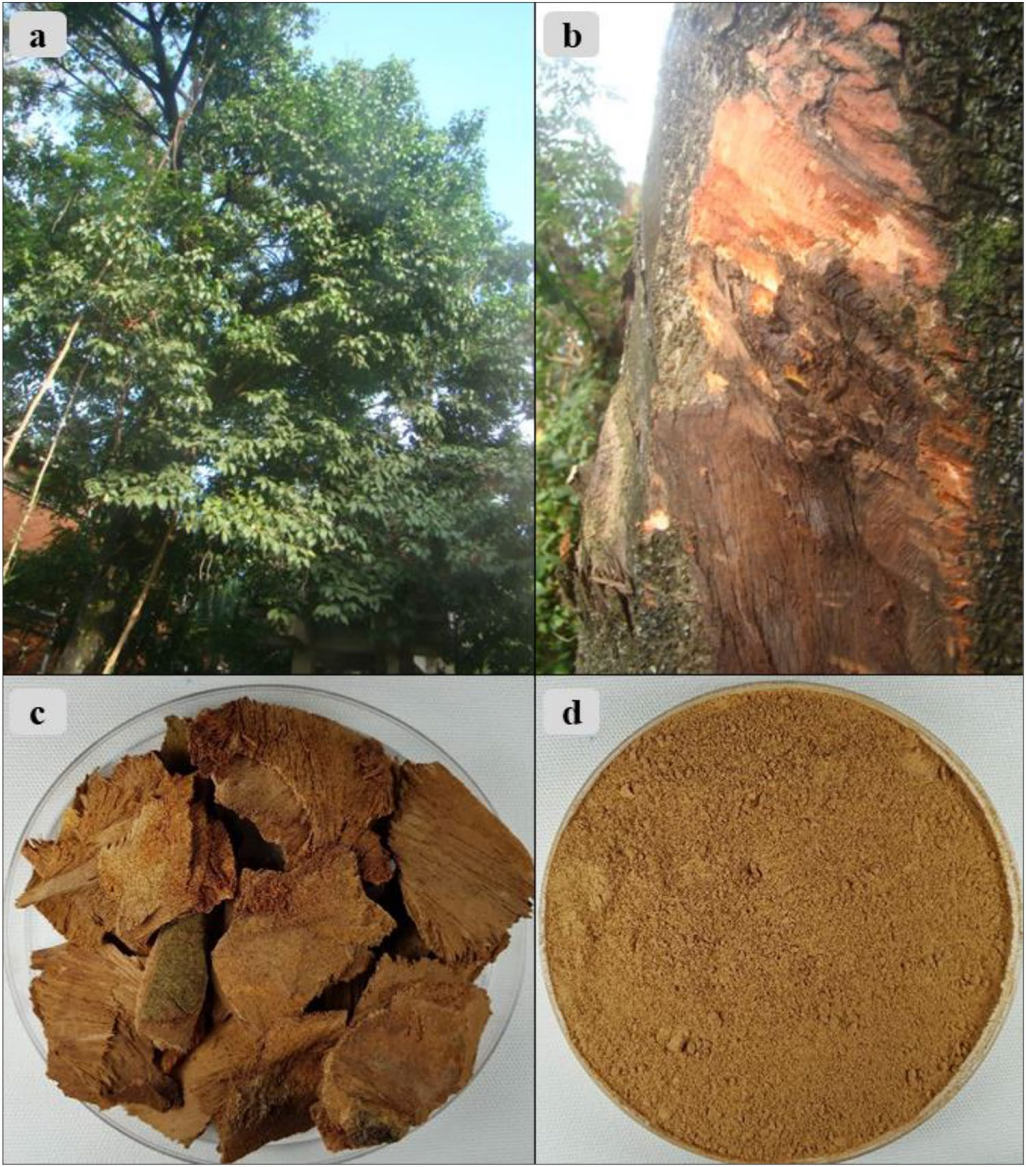
*Prunus africana* medicinal plant. (**a**)* P. africana* tree. (**b**) Stem of *P. africana* with part of its bark harvested for medicine purpose*.* (**c**) Harvested and dried *P. africana* stem bark. (**d**) Pulverized *P. africana* stem bark.

The study was conducted in accordance to the relevant institutional, national, and international guidelines and legislation. The stem bark of *P. africana* (1 kg) was obtained from *P. africana* tree (Fig. 1a,b) in the herbal garden of Natural Chemotherapeutics Research Institute, Ministry of Health, Uganda. The study material was identified by Dr. Sungyu Yang at Korea Institute of Oriental medicine (KIOM) and a voucher specimen (number KIOM201901022377) of the sample was deposited in the Korean Herbarium of Standard Herbal Resources (Index Herbarium code: KIOM) at KIOM, South Korea. The stem bark (Fig. 1c) was dried in an oven at 40 °C and then ground using a steel pulverizing machine (250G New Type Pulverizing Machine, Model RT-N04-2 V, Taiwan) to obtain a fine powder (Fig. 1d). 500 g of the fine powder sample was extracted by maceration using 1,500 ml of methanol. The extract was filtered using Whatman filter No. 1 after 24 h. and concentrated under a vacuum reduced pressure at 40 °C, 70 rpm, using an EYELA N-1200B (Tokyo Rikakikai Co. Ltd, Japan) efficient rotary evaporator. The concentrated extract was then vacuum dried and yielded 60 g of extract. The resultant dried extract was then used in the subsequent TRAP assay, cell viability assay, and experiments on the production of inflammatory factors.

### Gas chromatography-mass spectrometry (GC–MS) sample preparation and analysis

The *P. africana* bark sample was extracted in 100% methanol by sonication for 30 min. The extract was then prepared at 50 µg/L; filtered through a 0.2 μm syringe membrane filter from Whatman Ltd (Maidstone, UK) and subjected to GC–MS analysis. The analysis was performed in a 7890B GC–MS system (Agilent Technologies, Atlanta, GA, USA), coupled with a 7977B model mass detector (Agilent Technologies, Atlanta, GA, USA) using DB-5 MS capillary column (30 m × 0.25 mm × 0.25 μm). Chromatographic conditions were as follows: the extract (1 μL) was injected in split mode with a ratio of 1/20 at 250 °C; oven initial temperature was 50 °C and increased 110 °C during 5 min, followed by heating at a rate of 7 °C/min at 300 °C. The mass analyzer was set to scan from 30 to 600 amu. Peak identification was carried out by comparison of the experimental mass spectrum in the National Institute of Standards and Technology (NIST) and Wiley GC–MS libraries.

### Liquid chromatography time-of flight mass spectrometry (LC-TOF–MS) analysis

The *P. africana* bark sample was extracted and prepared as in “Gas chromatography-mass spectrometry (GC–MS) sample preparation and analysis” above. The LC-TOF–MS analysis was performed on an Agilent 1290 infinity II system coupled with an AB SCIEX Triple TOF 5600 mass spectrometer equipped with electrospray ionization. Gemini® C_18_ (250 mm × 4.6 mm i.d., 5 μm, Phenomenex, USA) was used for column separation. The column temperature was maintained at 40 °C, the flow rate was 1.0 ml/min, and the injection volume was 10 μl. The optimal mobile phase consisted of a linear gradient system of (A) 0.1% formic acid in water and (B) 0.1% formic acid in acetonitrile, 0–2 min, 3% B; 2–30 min, 3–35% B; 30–31 min, 35–50% B; 31–35 min, maintained 50% B; 35–40 min, 100% B; 40–45 min, maintained 100% B. Positive mode was applied in the ESI source with the following parameters: gas 1 = 50 psi, gas 2 = 50 psi, temperature = 500 °C, and 5500 V ion spray voltage with 30 psi curtain gas. Intact protonated molecular ions [M-Na]^+^ were detected via TOF–MS scan (100 psi declustering potential, 10 V collision energy, 100–2000 Da TOF MS scan range, and 250 ms accumulation time). Negative mode was applied in the ESI source with the following parameters: gas 1 = 50 psi, gas 2 = 50 psi, temperature = 500 °C, and − 4500 V ion spray voltage with 30 psi curtain gas. Intact protonated molecular ions [M-H]^−^ were detected via TOF–MS scan (− 100 psi declustering potential, − 10 V collision energy, 100–2000 Da TOF MS scan range, and 250 ms accumulation time).

### Inhibitory effect of *P. africana* on osteoclastogenesis

#### Cell culture and authentication

Mouse bone marrow macrophages (BMMs) were isolated from the tibia and femur of mice (male ICR mouse, 7 weeks old) by flushing with PBS as describe in previous study^20^. After over-night incubation in non-coated culture dish, non-attached cells, which were regarded as BMMs, were collected and cultured in proliferation medium [α-MEM medium with 10% FBS and macrophage-colony stimulating factor (M-CSF) (60 ng/mL)] for 7 days. To differentiate osteoclasts, BMMs (1 × 104 cells/well, 96-well plates) were cultured in α-MEM medium containing 10% FBS, M-CSF (60 ng/mL), and RANKL (100 ng/mL) for 4 days.

#### TRAP assay and BMM cell viability

This was performed following the previously described method^21^. The measurement of osteoclast TRAP activity was based on the generation of absorbance by incubating BMM cells with TRAP buffer (50 mM sodium tartrate, 0.12 M sodium acetate, pH 5.2) and *p*-nitrophenyl phosphate (1 mg/ml) for 15 min. For TRAP staining, the BMM cells were incubated with TRAP buffer containing naphthol AS-MX phosphate (0.1 mg/ml) and Fast Red Violet (0.5 mg/ml). The BMM cells were then cultured with *P. africana* methanolic extract at 0, 6.25, 12.5, 25, and 50 µg/ml concentrations and 50 μM of β-sitosterol, chlorogenic acid, catechin and naringenin each in the presence of RANKL for 6 days. The osteoclast TRAP activity was determined using a colorimetric assay with *p-*nitrophenyl phosphate as a substrate. The cell viability was determined using Cell Counting Kit-8 (CCK) (WST-8/CCK8; Dojindo), according to the manufacturer’s instructions. For the measurement of cell viability, cells were plated in 96-well plates and treated with *P. africana* extracts concentrations as above for 24 h. After incubating with the CCK solutions and the cells for 1 h, the absorbance was measured at 450 nm using a microplate reader (Versa Max) and results were presented as a percentage of the vehicle control.

### Nitrite (NO) assay and RAW 264.7 cell viability

Murine macrophage RAW 264.7 cells were cultured in DMEM medium supplemented with 100 U/mL of penicillin, 100 ug/mL of streptomycin, and 10% heat-inactivated FBS. The nitrite concentration in the supernatant from cultured cells was analyzed using the Griess reaction test. RAW 264.7 cells were plated at a density of 5 × 10^4^ cells/mL in 96-well culture plates, pre-incubated with samples (*P. africana* methanolic extracts (150 μg/mL), β-sitosterol, chlorogenic acid, catechin and naringenin (100 μM each) for 3 h, and stimulated with LPS (200 ng/ml) for 24 h. Griess reagent (1% sulfanilamide, 0.1% N-1-napthylethylenediamine dihydrochloride, and 2.5% phosphoric acid) was mixed with an equal volume of cell supernatant, and absorbance was measured at 570 nm using the ELISA reader. Sodium nitrite was used as a standard. Dexamethasone was used as a positive control (40 or 80 μM).

### Enzyme-linked immunosorbent assay (ELISA)

The concentrations of the inflammatory cytokines IL-1β (R&D, USA) and IL-6 (MyBiosource, USA) in culture supernatant was determined using ELISA antibody kits following the manufacturer1s protocol (MyBiosource, USA). RAW264.7 cells were grown in 96-well culture plates at a density of 5 × 10^4^ cells/mL, pre-incubated with samples for 3 h, and stimulated with LPS for 6 h (IL-1β) or 24 h (IL-6). The cytokines produced in each sample were calculated from standard curves using known concentrations of recombinant cytokines for each ELISA antibody kit.

### Hepatotoxicity assay in zebrafish (*Danio rerio*) larvae

This was performed following the previously described method^21^. All methods were carried out in accordance with relevant guidelines and regulations. All experimental protocols were approved by Korea Research Institute of Chemical Technology research ethics committee and conducted in compliance with the ARRIVE guidelines. Zebrafish larvae were used for this study as previously described^22^. At 96 h post-fertilization (hpf), the larvae were transferred to a transparent 24-well plate (N = 10/well) with 1 ml of embryonic medium. The larvae were then exposed to increasing concentrations of 25, 50, 100, and 200 µg/ml of *P. africana* methanolic and ethanolic extracts and water extract from 90 to 120 hpf. Dimethyl sulfoxide (DMSO) was used as a negative control while 5 μM of tamoxifen (Sigma-Aldrich, St. Louis, MO, USA) was used as a positive control. To obtain images, the larvae were anesthetized in tricaine (Sigma-Aldrich), mounted in 3% methyl cellulose (Sigma-Aldrich), and observed under a Leica MZ10 F stereomicroscope equipped with a Leica DFC425 camera and Leica application Suite software (version 4.5).

### Statistical analysis

Data were represented as the mean ± standard deviation. Statistical significance between groups was analyzed using the Student’s t-test and *p*-values < 0.05 were considered statistically significant**.**

## Results

### GC/MS analysis

The GC/MS analysis of the *P. africana* extract was based on mass spectra, retention times, and quality ratio analysis revealed the presence of 32 components (Table 1) including 3-Furanmethanol (1), Dihydroxyacetone (2), Benzoic acid, methyl ester (3), 4H-Pyran-4-one,2,3-dihydro-3,5-dihydroxy-6-methyl- (4), benzoic acid (5), Catechol (6), 4-Vinylphenol (7), 5-Hydroxymethyl-2-furaldhyde (8), Isosorbide (9), Phenol, 2,6-dimethoxy- (10), 4-Hydroxy-3-methoxybenzaldehyde (11), 3,4-Altrosan (12), Mandelamide (13), Vanillic acid (14), Benzenepropanol, 4-hydroxy-3-methoxy- (15), Benzaldehyde, 4-hydroxy-3,5-dimethoxy- (16), 4-(hydroxymethyl)-2,6-dimethoxyphenol (17), (E)-4-(3-Hydroxyprop-1-en-1-yl)-2-methoxyphenol (18), 6-Hydroxy-5-trifluoromethylcyclohexa-1,3-diene (19), Benzoic acid, 4-hydroxy-3,5-dimethoxy- (20), Isopropyl myristate (21), Sorbitol (22), n-Hexadecanoic acid (23), 9,12-Octadecadienoic acid (Z,Z)- (24), Oleic acid (25), Octadecanoic acid (26), Benzyl, beta-d-glucoside (27), 9-Octadecenamide, (Z)- (28), (R)-alpha-(beta-D-glucopyranosyloxy)benzene-acetonitrile (29), 13-Docosenamide, (Z)- (30), Squalene (31), Beta-Sitosterol (32).

**Table 1:** Phytochemical components identified in the stem bark of *Prunus africana* methanolic extract by GC/MS analysis.

Peak no	Identified compound	*t*_*R*_ (min)	% of total	Quality (%)
1	3-Furanmethanol	5.96	0.39	93
2	Dihydroxyacetone	7.01	2.22	74
3	Benzoic acid, methyl ester	12.83	0.46	91
4	4H-Pyran-4-one,2,3-dihydro-3,5-dihydroxy-6-methyl-	13.95	1.13	95
5	benzoic acid	14.68	14.02	91
6	Catechol	15.17	4.74	91
7	4-Vinylphenol	15.61	0.77	72
8	5-Hydroxymethyl-2-furaldhyde	15.81	0.31	89
9	Isosorbide	17.10	0.98	93
10	Phenol, 2,6-dimethoxy-	18.39	0.41	94
11	4-Hydroxy-3-methoxybenzaldehyde	19.30	0.41	93
12	3,4-Altrosan	20.84	3.30	76
13	Mandelamide	21.54	0.40	93
14	Vanillic acid	22.13	0.93	95
15	Benzenepropanol, 4-hydroxy-3-methoxy-	23.61	2.14	92
16	Benzaldehyde, 4-hydroxy-3,5-dimethoxy-	23.83	0.75	91
17	4-(hydroxymethyl)-2,6-dimethoxyphenol	24.57	0.17	87
18	(E)-4-(3-Hydroxyprop-1-en-1-yl)-2-methoxyphenol	25.03	0.18	91
19	6-Hydroxy-5-trifluoromethylcyclohexa-1,3-diene	25.98	3.65	59
20	Benzoic acid, 4-hydroxy-3,5-dimethoxy-	26.13	0.29	96
21	Isopropyl myristate	26.31	0.40	99
22	Sorbitol	27.95	0.05	91
23	n-Hexadecanoic acid	28.21	4.95	99
24	9,12-Octadecadienoic acid (Z,Z)-	30.51	0.20	99
25	Oleic acid	30.58	0.76	98
26	Octadecanoic acid	30.87	0.47	97
27	Benzyl, beta-d-glucoside	31.90	0.32	87
28	9-Octadecenamide, (Z)-	33.35	1.95	99
29	(R)-alpha-(beta-D-glucopyranosyloxy) benzene-acetonitrile	35.10	6.60	83
30	13-Docosenamide, (Z)-	37.88	6.49	99
31	Squalene	38.38	1.09	99
32	Beta-Sitosterol	43.55	8.37	99

### LC-TOF–MS analysis

Based on the chemical profiling by LC-TOF–MS analysis, the various phytochemicals were detected from the *P. africana* extract. In the positive ion mode, as a result of analysis using retention index libraries, 65 peaks with a library score over 90% were identified from the *P. africana* extract (Supplementary Table Y). Among them, 24 components showed reliable mass (over 98% of library score) in the *P. africana* extract, especially, 7 components (Astragalin, Chlorogenic acid, Coproporphyrin I, Hyperin, Luteoloside, Mesoporphyrin IX and Naringenin), showed the accurate mass according to result of 100% library score (Table 2). In the negative ion mode, 72 peaks with a library score over 90% were identified from the *P. africana* extract (Supplementary Table Z). Among them, 29 components showed the reliable mass (over 98% of library score) in the *P. africana* extract, especially, 5 components (Pedunculoside, Luteoloside, Hexadecanedioic acid, Guanosin, Betulonic acid and Naringenin), showed the accurate mass according to result of 100% library score (Table 2). In the present study, among the identified primary and secondary metabolic components, Catechin showed the largest peak area among the identified primary and secondary metabolic components in the *P. africana* extract (Supplementary Table Y and Z).

**Table 2:** Phytochemical components identified in the stem bark of *P. africana* methanolic extract by LC-TOF–MS analysis.

No	Name	Mass ([M-Na]^+^)	Founded mass	RT (min)	Founded RT (min)	Peak area	Library score (%)
1	Astragalin	449.1083	449.1083	23.25	23.22	1129.49	100.00
2	Chlorogenic acid	355.1024	355.1024	13.16	13.14	2289.52	100.00
3	Coproporphyrin I	655.4932	655.4961	1.19	1.32	1974.83	100.00
4	Hyperin	465.1033	465.1033	23.12	23.12	3329.70	100.00
5	Luteoloside	449.1787	449.1787	22.56	22.56	4448.29	100.00
6	Mesoporphyrin IX	567.2814	567.2809	42.57	42.56	1217.89	100.00
7	Naringenin	273.0762	273.0762	33.74	33.73	6559.30	100.00

No	Name	Mass ([M-H]–)	Founded mass	RT (min)	Founded RT (min)	Peak area	Library score (%)
1	Pedunculoside + HCOOH	695.3693	695.3696	34.39	34.38	20,498.21	100.00
2	Luteoloside	447.0696	447.0695	23.18	23.21	1513.48	100.00
3	Hexadecanedioic acid	285.1910	285.1909	40.49	40.48	4854.99	100.00
4	Guanosine	282.0683	282.0684	6.66	6.65	7969.05	100.00
5	Betulonic acid	453.2775	453.2776	42.97	42.97	1209.40	100.00

### TRAP assay and BMM viability

**Figure 2:**
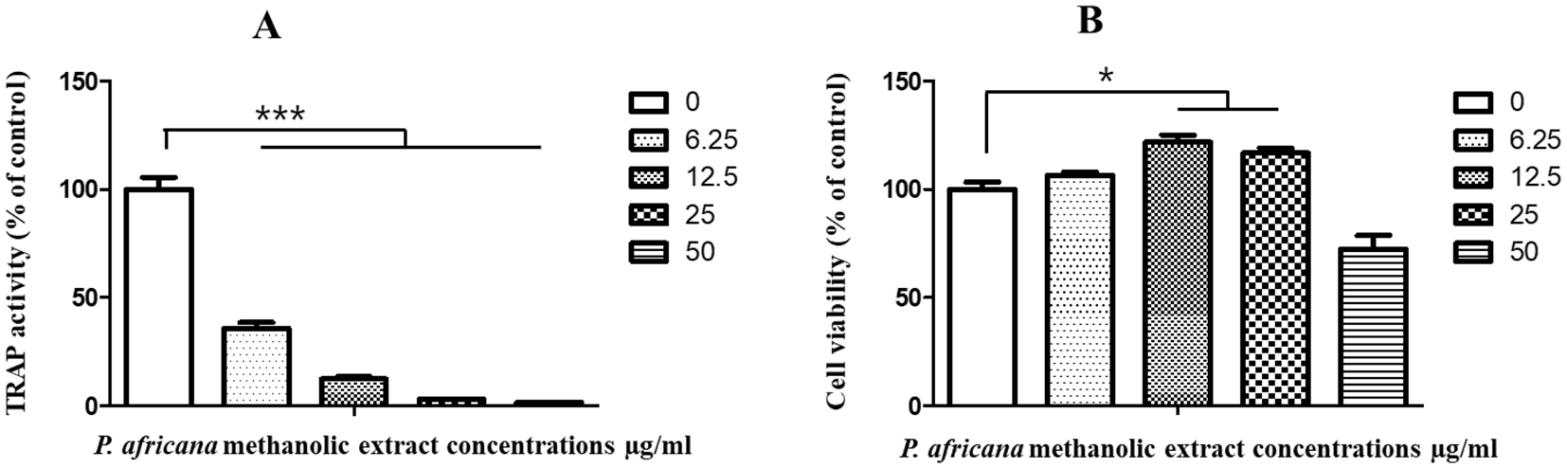
Effect of *P. africana* on TRAP activity in BMM. The BMM were cultured with *P. africana* bark extracts in the presence of RANKL for 6 days and TRAP activity of osteoclasts measured by colorimetric assay using p-nitrophenyl phosphate as a substrate. Cell viability was determined using Cell Counting Kit-8 following manufacturer’s instruction. *p < 0.05 and ***p < 0.001.

TRAP activity was significantly (*p* < 0.001) inhibited compared to the control at concentrations of 6.25, 12.5, 25, and 50 µg/ml methanolic *P. africana* stem bark extracts (Fig. 2A).

*Prunus africana* methanolic stem bark extract had a significant (*p* < 0.05) simulative effect at concentrations of 12.5 and 25 µg/ml on the cell viability of BMM cells (Fig. 2B) compared to the control. However, at a higher concentration of 50 µg/ml the methanolic stem bark extract, the cell viability reduced to 70% compared to the control. The high viability of the BMM cells may indicate the non-cytotoxicity of *P. africana* bark.

**Figure 3:**
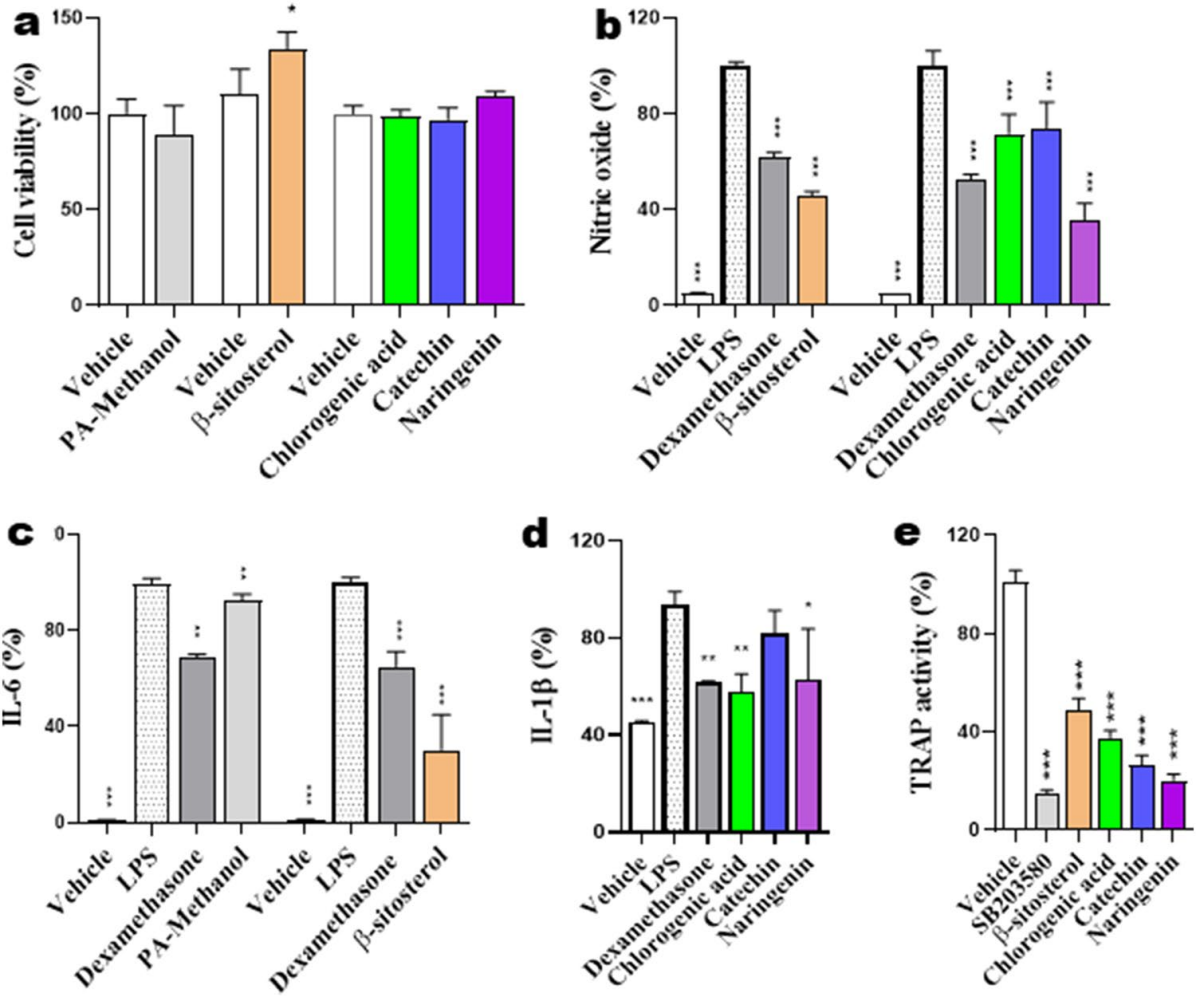
Effects of samples (PA-methanol, β-sitosterol, chlorogenic acid, catechin, and naringenin) on (**a**) cell viability, (b-d) the production of inflammatory factors (nitric oxide, IL-6, or IL-1β), or (**e**) TRAP activity. After 3 h pre-incubation of samples, RAW 264.7 cells were treated with LPS for 6 to 48 h depending on the assay condition. (**a**) Cell viability was measured using a CCK assay. (**b**) Nitric oxide content in the medium was determined using Griess reagent assay; (**c**) IL-6 and (**d**) IL-1β cytokine levels in the medium were measured using ELISA kit. (**e**) TRAP activity was examined by using TRAP buffer containing naphthol AS-MX phosphate. Positive control: 40 or 80 μM dexamethasone. As a control, cells were incubated with the vehicle alone. * p < 0.05, ** p < 0.01 and *** p < 0.001.

The effects of β-sitosterol, chlorogenic acid, catechin and naringenin (50 μM each) on RANKL-induced TRAP activity representing osteoclastogenesis were evaluated. We found that these compounds significantly inhibited RANKL-induced TRAP activity without showing cell toxicity (Fig. 3).

### Inhibitory effect on NO production

Since NO production is correlated with various inflammatory diseases, we determined the suppressive effects of samples (*P. africana* (PA) methanolic extract, β-sitosterol, chlorogenic acid, catechin and naringenin) on NO levels in RAW264.7 cells via LPS stimulation. To determine NO levels in the supernatant, cells were pre-treated with samples for 3 h, followed by stimulation with LPS for 24 h, and then measured using Griess reagent. As the positive control, dexamethasone showed strong suppressive effect on NO secretion upon LPS stimulation. All the investigated samples dramatically inhibited NO production after LPS stimulation (Fig. 3b). All samples did not significantly affect cell viability and β-sitosterol that increased it (Fig. 3a).

### Inhibitory effect on IL-6 and IL-1β levels

The effects of samples on inflammatory cytokine, IL-6, secretion in macrophages were evaluated using enzyme-linked immunosorbent assay (ELISA). IL-6 secretion was significantly inhibited by PA-methanol (p < 0.001) and β-sitosterol (p < 0.0001) (Fig. 3c). Chlorogenic acid and naringenin, but not catechin, significantly inhibited LPS-induced IL-1β level as shown in Fig. 3d.

### Hepatotoxicity in zebrafish larvae

**Figure 4:**
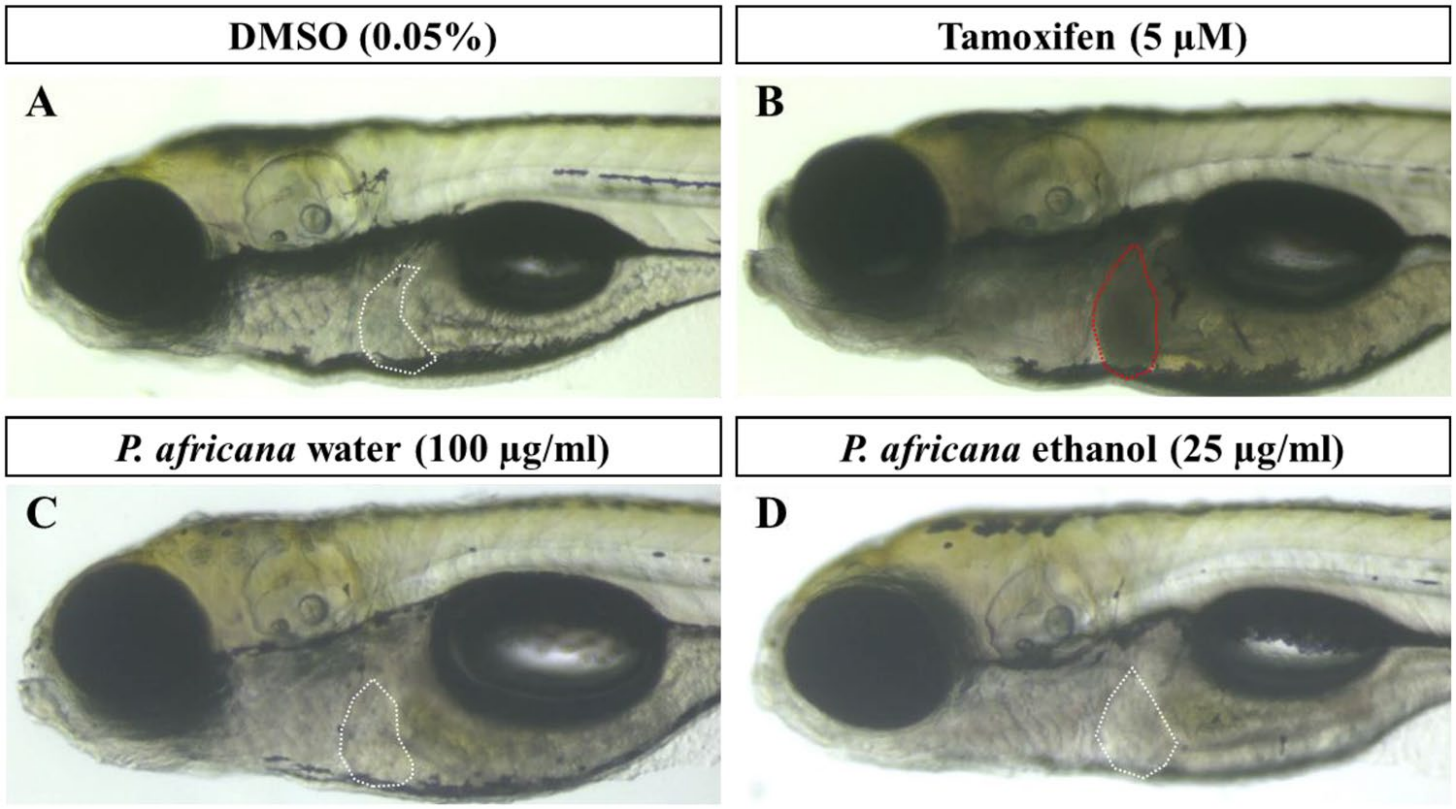
Hepatotoxicity assay in zebrafish larvae at 120 hpf. (**A**) DMSO as a negative control did not induce hepatotoxicity. (**B**) Tamoxifen induced liver cell death (shown by red arrow). (**C**) 100 µg/ml *P. africana* water did not induce hepatotoxicity. (**D**) 25 µg/ml *P. africana* ethanol did not induce hepatotoxicity.

In this study, the zebrafish larvae exposure was done from 96 to 120 hpf and those exposed to DMSO showed no liver cell death (shown by white dash line) (Fig. 4A) but tamoxifen treatment resulted in liver cell death (shown by red dash line) (Fig. 4B). At a concentration of 50 and 100 µg/ml water extract of *P. africana*, 30% and 10% of the zebrafish larvae survived at 120 hpf respectively and death of hepatocytes was not observed in them (Fig. 4C). However, at a higher concentration of 200 µg/ml *P. africana* water extract, 100% larvae mortality was observed before 120 hpf. At a concentration of 25 µg/ml ethanolic extract, 50% of the zebrafish larvae survived and hepatocytes death was not observed in them at 120 hpf (Fig. 4D). However, at higher concentrations of 50 and 100 µg/ml, 100% larvae mortality rate was observed at 120 hpf. 100% larvae mortality was observed for larvae exposed to *P. africana* bark methanolic extracts at various concentrations of 25, 50, and 100 µg/ml before the 120 hpf.

## Discussion

TRAP is a specific and reliable cytochemical marker used as a measure of activated macrophages^23^. The bone marrow macrophages play a major role in the activation and formation of osteoclasts and hence important for the pathogenesis of osteoporosis^5^. Osteoporosis reflects increased osteoclast function relative to that of osteoblasts and hence the pharmacological arrest of osteoclasts is a mainstay in the treatment of systemic bone loss^24^. TRAP activity is an important cytochemical marker of osteoclasts and its concentration in the serum is utilized as a biochemical and histochemical marker of osteoclast function and degree of bone resorption^25^. Therefore, as a measure of osteoclast number and bone resorption, TRAP plays a vital role in osteoporosis diagnosis and prognosis^23^. And as evidenced in the previous study, the suppressing of TRAP pathway prevented ovariectomy-induced osteoporosis *in vivo*^26^. Therefore, the significant (*p* < 0.001) inhibition compared to the control at concentrations of 6.25, 12.5, 25, and 50 µg/ml methanolic *P. africana* stem bark extracts is an indication of *P. africana* anti-osteoporosis effects. Additionally, chlorogenic acid, catechin, naringenin and β-sitosterol that are present in *P. africana* stem bark have been demonstrated in our study to remarkably inhibit RANKL-induced TRAP activity explaining indeed the antiosteoporotic potential of the plant stem bark.

The anti-osteoporosis activity of *P. africana* bark extracts may be attributed to some of the compounds present in it including astragalin, hyperin, luteoloside, mesoporphyrin, naringenin, chlorogenic acid, β-sitosterol and catechin (Tables 1 and 2, and supplementary Tables Yand Z). Additionally, previous studies have also showed that *P. africana* stem bark is indeed rich in these compounds with anti-osteoporosis activity (Table 3). Astragalin demonstrated estrogenic anti-osteoporosis activity and significantly increased proliferation in osteoblastic cells (UMR-106)^27^. In another study, Astragalin promoted differentiation in MC3T3-E1 osteoblastic cells through activation of MAPK and BMP pathways and promoted in vivo bone formation^28^. Further, a number of other studies have showed anti-osteoporosis activity of Astragalin^10,29,30^, and its presence in *P. africana* stem bark as confirmed in our study shows that it indeed contributes to the anti-osteoporosis activity of the plant stem bark. Hyperin was one of the major 3 chemical compounds in *Cuscuta chinensis* that showed anti-osteoporosis effects^29^. In fact in their study, Tao, et al.^29^ ascribed the anti-osteoporosis effect of *C. chinensis* to hyperin as a result of its high positive correlation to anti-osteoporosis activity. Hyperin was also reported to markedly increase alkaline phosphatase (ALP) activity in osteoblast cells^31^.

**Table 3:** Summary of the previous studies on anti-osteoporosis effects of some of the compounds identified in *P. africana.*

S/No	Phytochemical compounds	Compound structure	Anti-osteoporosis effects	Reference
(a)	Vanillic acid		Improves bone mineral density and bone mineral content; Protects the trabecular structure from being degraded by osteoclasts	^43,44^
(b)	Sorbitol		Retards bone resorption	^45^
(c)	Octadecanoic acid (Stearic acid)		Inhibits osteoclastogenesis in vitro	^46^
(d)	β-sitosterol		Inhibits osteoporosis through retardation of acute inflammation	^47^

Luteoloside, a natural compound is known to suppress activity of osteoclasts thus could potentially be used for treating bone metabolism disorders including osteoporosis^32^. Luteoloside in a previous study was showed to possess strong inhibition against LPS induced osteolysis in an in vivo study^32^. Further, it was also demonstrated to suppress differentiation of RANKL-induced osteoclast and decrease bone resorption tendency dose dependently^32^. The anti-osteoclastic and anti-resorptive actions of luteoloside were not only through blockage of NFATc1 activity and debilitation of RANKL-mediated Ca^2+^ signaling but also through MAPK and NF-κB pathways^32^. Mesoporphyrin, a porphyrin derivative has been reported to possess anti-inflammatory activity through inhibiting IL-6 production^33^. Since IL-6 potently activates osteoclasts and is responsible for bone resorption^34^, mesoporphyrin IX may thus possess anti-osteoporosis activity through inhibition of IL-6 production.

Naringenin has been reported to significantly inhibit osteoclastogenesis with inhibitions of up to 96 ± 1% at 50 μg/ml^35^. Naringenin has also been demonstrated to not only significantly inhibit secretion of monocyte chemoattractant protein-1, interleukin (IL)-1α and IL-23 but also markedly decrease release of a bone resorption activity indicator, helical peptide 620–633, thus greatly inhibiting osteoclastic bone resorption and human osteoclastogenesis^35^. La, et al.^35^ indicates that naringenin could be used to treat bone-related diseases such as osteoporosis. In the current study, naringenin was observed to significantly inhibit LPS-induced NO production to a level even greater than the effect of dexamethasone (positive control) and also remarkably suppress IL-1β generation. Since increased NO level inhibits growth and differentiation of osteoblasts^36^, and IL-1β stimulates bone resorption^37^, naringenin suppression of NO and IL-1β production signifies that it is important in preventing osteoporosis. In another study, naringenin was showed to significantly promote osteogenic differentiation^38^. Naringenin being present in the stem bark of *P. africana* contributes to its anti-osteoporosis activity. Catechin was identified as possessing the strongest osteogenic effects from a batch of herbal ingredients used in traditional Chinese medicine using human mesenchymal stem cells (hMSCs)^39^. Catechin was reported to increase the activity of ALP, deposition of calcium and Runx2 mRNA expression among others^39^. It was thus proposed to enhance osteogenesis through increasing protein phosphatases 2A (PP2A) level inhibiting extracellular signal-regulated kinase (ERK) signaling in hMSCs^39^. In another study, catechin rich extract was demonstrated to promote formation and enhance survival of osteoblasts and inhibiting the activity and growth of osteoclasts^40^. Similar to our findings, catechin in previous studies has been documented to suppress NO production in LPS stimulated macrophages^41,42^. Thus, our study suggests that catechin exerts antiosteroporotic effect by eliminating the inhibitory effect of NO on growth and differentitation of osteoblasts. The presence of catechin in the stem bark of *P. africana* enhances its anti-osteoporosis potential.

Vanillic acid has been reported to exhibit anti-osteoporotic activity by inhibitory effects on bone resorption^48^; improving bone mineral density and bone mineral content and as well as biomechanical stability^43^ and protecting trabecular structure from degradation by osteoclasts in ovariectomized postmenopausal mice^44^. Sorbitol; a sugar alcohol with a sweet taste has been observed to retard bone resorption in Sprague Dawley male rats^45^. The positive effects on osteoporosis prevention by fruits including that of *Prunus domestica* and *Prunus salicina* may partly be due to the presence of sorbitol compound in them^1^. Recent studies suggest that inflammation is one of the key factors that influence bone turnover, leading to osteoporosis^3^. Therefore, the potent anti-inflammatory activity of β-sitosterol^47^; a compound present in *P. africana* stem bark may further explain its anti-osteoporosis activity. Stearic acid and oleic acid have been reported to inhibit osteoclastogenesis in bone marrow cultures and RAW264.7 cells^46^. Chlorogenic acid was observed to improve the bone quality by modifying the bone mineral density and trabecular microarchitecture in an ovariectomy rat model^49^. Furthermore, chlorogenic acid was also observed to promote proliferation of osteoblast precursors and osteoblastic differentiation in ovariectomized rats^50^. In the present study, chlorogenic acid exhibited significant inhibition of LPS-induced NO production and secretion of IL-1β. Interleukin-1 is a very powerful stimulator of bone resorption and is well known to inhibit bone formation^51^. The significant inhibition of IL-1β and NO production by chlorogenic acid indicates that it plays an important role in preventing and treating osteoporosis. In addition, our study showed that chlorogenic acid had a significant inhibition of RANKL-induced TRAP activity giving further evidence of its antiosteoporotic potential.

In light of these results, the inhibition of the TRAP activity by *P. africana* bark extracts may therefore be due to these different important compounds in it including chlorogenic acid, catechin, naringenin and β-sitosterol. These findings therefore provide valuable insight in to the anti-osteoporosis potential of *P. africana*.

Excess production of NO in the body system plays a vital part in pathogenesis of inflammatory diseases including osteoporosis^21^. It has been reported previously that increased production of NO is a contributing factor to osteoporosis pathogenesis^21,52^. Thus, a potential therapeutic pathway for managing the disease is through suppression of NO production as indicated by Komakech, et al.^21^. In this study, the *P. africana* methanolic extract (each 150 µg/ml) significantly (p < 0.0001) inhibited NO production actually more than the positive standard, dexamethasone (40 µM). The chemical compounds, chlorogenic acid, catechin, naringenin and β-sitosterol (100 µM each) in RAW264.7 cells also significantly suppressed LPS-induced NO production. Studies have reported that high NO concentrations have great inhibitory effects on growth and differentiation of osteoblasts^36,53–55^. This has been suggested to be partly as a result of NO pro-apoptotic effects on osteoblasts^56^. The significant inhibition of LPS-induced NO production by PA extracts and its chemical constituents clearly demonstrates its antiosteoporotic potential by eliminating the inhibitory effects of NO to growth and differentiation of osteoblasts. Chlorogenic acid, catehin, naringenin and β-sitosterol did not show toxic effects.

In our current study, PA-methanolic extract (150 µg/ml) and β-sitosterol (100 µM) just like the positive control (dexamethasone [40 µM and 80 µM]) in RAW264.7 cells were observed to significantly inhibit LPS-induced IL-6 secretion. This therefore suggests that methanol extracted phytochemical compounds from *P. africana* stem bark including β-sitosterol exhibited antiosteoporotic potential by inhibiting the production of IL-6 within the bone microenvironment. IL-6 is known to potently activate osteoclasts and is responsible for bone resorption^34^. Pro-inflammatory cytokines notably IL-6 are vital in normal processes of bone remodeling and pathogenesis of osteoporosis in elderly persons and during perimenopause^34^. Production of IL-6 induces eminent lytic lesions along with diffuse osteoporosis typical of the disease^34^. Before menopause, estrogen in bone marrow regulates the expression of most notably IL-6^34^. IL-6 levels are known to increase with age in not only humans but monkeys and mice and in the trend with osteoporosis^34^.

Liver toxicity from herbal and dietary supplements is a common phenomenon and is a leading cause of a number of underlying liver diseases^57^. Osteoporosis is a frequent complication in patients with liver complications^58^. Indeed, decreased trophic factors such as insulin growth factor in the liver due to liver toxicity or chronic diseases including diabetes my result in osteoblast dysfunction^19^. Therefore, ensuring a healthy liver is fundamental in maintaining a balanced body biological processes including prevention of bone loss. Zebrafish larvae is an important model system for the evaluation of the liver toxicity when an organism is exposed to a toxicant^59^. Liver organogenesis in zebrafish initiates at 30 hpf on the left-hand side of the embryo and an enlarged liver bud connects with the intestine and functionally matures until 72 hpf^60^. Treatment of the zebrafish larvae with liver toxicants such as tamoxifen reduces liver transparency due to the liver cell death^61^. In this study, 200 µg/ml water extract and 25 µg/ml methanolic extract exhibited maximum concentrations for the acute toxicity. Thus, tests at 100 µg/ml water extract and 25 µg/ml ethanolic extract was used to determine hepatotoxicity in zebrafish larvae. And although each experimental group showed larval mortality, the *P. africana* extracts did not induce hepatotoxicity because living larvae did not show liver-specific cell death. Considering that the extraction method can influence the composition of extracts^62^, this may explain difference in the mortality rate of the zebrafish larvae in the methanolic and water extracts of the *P. africana* stem bark. Previous studies also showed that *P. africana* bark extract administered at 1000 mg/kg body weight had no visible deleterious effects on BALB/c mice^63^ and showed mild hepatotoxicity and nephrotoxicity in Sprague–Dawley rats^64^. These observations therefore showed that the stem bark of *P. africana* its non-toxic within a low dose range.

## Conclusions

The macrophage-dependent anti-osteoporosis activity of *P. africana* bark may be attributed to the synergistic action of the various phytochemicals in its stem bark including chlorogenic acid, catehin, naringenin, vanillic acid, sorbitol, octadecanoic acid (stearic acid), and β-sitosterol. NO production was significantly inhibited (all p < 0.0001) by *P. africana* methanolic, chlorogenic acid, catehin, naringenin, and β-sitosterol. IL-6 secretion was significantly inhibited by *P. africana* methanolic extract (p < 0.0001) and β-sitosterol (p < 0.0001) and in addition, chlorogenic acid and naringenin remarkably inhibited IL-1β production. All samples displayed significant inhibition of RANKL-induced TRAP activity. Although the methanolic extract of *P. africana* bark exhibited potent anti-osteoporosis activity, we recommend that future studies should carry out isolation of the individual chemicals or group of chemicals that are/is responsible for its anti-osteoporosis activity*.* Nonetheless, this study has demonstrated that *P. africana* bark extracts have no overt hepatotoxic effects in zebrafish larvae at a given dose range and offers a basis for future studies and medicine development with anti-osteoporosis therapeutic application.

## Supplementary Information

Supplementary Information.

## Data Availability

The data for this current study are available from the corresponding author upon reasonable request.

## References

[1] Higgs J, Derbyshire E, Styles K (2017). Nutrition and osteoporosis prevention for the orthopaedic surgeon: A wholefoods approach. EFORT Open Rev..

[2] An J (2016). Natural products for treatment of osteoporosis: The effects and mechanisms on promoting osteoblast-mediated bone formation. Life Sci..

[3] Ginaldi L, Mengoli LP, De Martinis M (2009). Handbook on Immunosenescence.

[4] Sözen T, Özışık L, Başaran NÇ (2017). An overview and management of osteoporosis. Eur. J. Rheumatol..

[5] Yang D-H, Yang M-Y (2019). The role of macrophage in the pathogenesis of osteoporosis. Int. J. Mol. Sci..

[6] Hirano T (1998). Interleukin 6 and its receptor: Ten years later. Int. Rev. Immunol..

[7] Hirano T (2021). IL-6 in inflammation, autoimmunity and cancer. Int. Immunol..

[8] Ota N (2001). A nucleotide variant in the promoter region of the interleukin-6 gene associated with decreased bone mineral density. J. Hum. Genet..

[9] Suvarna V (2018). Bone health and natural products-an insight. Front. Pharmacol..

[10] Jia M (2012). Potential antiosteoporotic agents from plants: a comprehensive review. Evid.-Based Complement. Altern. Med..

[11] Evelyn SS, Chitra V (2019). Medicinal plants for the treatment of postmenopausal osteoporosis. Biomed. Pharmacol. J..

[12] Rates SMK (2001). Plants as source of drugs. Toxicon.

[13] Jimu L (2011). Threats and conservation strategies for the African cherry (Prunus africana) in its natural range-A review. J. Ecol. Nat. Environ..

[14] Nyamai D (2015). Phytochemical profile of Prunus africana stem bark from Kenya. J. Pharmacogn. Nat. Products.

[15] Komakech R, Kang Y (2019). Ethnopharmacological potential of African cherry [Prunus africana]. J. Herb. Med..

[16] Smith MR (2002). Osteoporosis during androgen deprivation therapy for prostate cancer. Urology.

[17] Daniell HW (2000). Progressive osteoporosis during androgen deprivation therapy for prostate cancer. J. Urol..

[18] Amadi CN, Orisakwe OE (2018). Herb-induced liver injuries in developing nations: An update. Toxics.

[19] Kalaitzoglou E, Popescu I, Bunn RC, Fowlkes JL, Thrailkill KM (2016). Effects of type 1 diabetes on osteoblasts, osteocytes, and osteoclasts. Curr. Osteoporos. Rep..

[20] Lee J-H (2009). Trolox prevents osteoclastogenesis by suppressing RANKL expression and signaling. J. Biol. Chem..

[21] Komakech R (2020). In vitro antiosteoporosis activity and hepatotoxicity evaluation in zebrafish larvae of bark extracts of Prunus jamasakura medicinal plant. Evid.-Based Complement. Altern. Med..

[22] Westerfield, M. (University of Oregon Press Eugene, OR, 2000).

[23] Janckila AJ, Yam LT (2009). Biology and clinical significance of tartrate-resistant acid phosphatases: New perspectives on an old enzyme. Calcif. Tissue Int..

[24] Teitelbaum SL (2007). Osteoclasts: What do they do and how do they do it?. Am. J. Pathol..

[25] Blumer MJ (2012). Role of tartrate-resistant acid phosphatase (TRAP) in long bone development. Mech. Dev..

[26] Yıldırım M (2020). White tea reduced bone loss by suppressing the TRAP/CTX pathway in ovariectomy-induced osteoporosis model rats. Cells Tissues Organs.

[27] Yang L, Chen Q, Wang F, Zhang G (2011). Antiosteoporotic compounds from seeds of Cuscuta chinensis. J. Ethnopharmacol..

[28] Liu L (2019). Astragalin promotes osteoblastic differentiation in MC3T3-E1 cells and bone formation in vivo. Front. Endocrinol..

[29] Tao Y, Chen L, Pan M, Zhu F, Yan J (2021). Tracing anti-osteoporosis components from raw and salt-processed semen of Cuscuta chinensis by employing a biochemometrics strategy that integrates ultrasonic-assisted extraction, quantitation, efficacy assessment in zebrafish, and grey relationship analysis. J. Sep. Sci..

[30] Riaz A (2018). Astragalin: A bioactive phytochemical with potential therapeutic activities. Adv. Pharmacol. Sci..

[31] Yang HM, Shin H-K, Kang Y-H, Kim J-K (2009). Cuscuta chinensis extract promotes osteoblast differentiation and mineralization in human osteoblast-like MG-63 cells. J. Med. Food.

[32] Song F (2018). Luteoloside prevents lipopolysaccharide-induced osteolysis and suppresses RANKL-induced osteoclastogenesis through attenuating RANKL signaling cascades. J. Cell. Physiol..

[33] Takaoka Y, Matsuura S, Boda K, Nagai H (1999). The effect of mesoporphyrin on the production of cytokines by inflammatory cells in vitro. Jpn. J. Pharmacol..

[34] Ershler WB, Harman SM, Keller ET (1997). Immunologic aspects of osteoporosis. Dev. Comp. Immunol..

[35] La V, Tanabe S, Grenier D (2009). Naringenin inhibits human osteoclastogenesis and osteoclastic bone resorption. J. Periodontal Res..

[36] Ralston SH, Todd D, Helfrich M, Benjamin N, Grabowski PS (1994). Human osteoblast-like cells produce nitric oxide and express inducible nitric oxide synthase. Endocrinology.

[37] Amano S, Kawakami K, Iwahashi H, Kitano S, Hanazawa S (1997). Functional role of endogenous CD14 in lipopolysaccharide-stimulated bone resorption. J. Cell. Physiol..

[38] Wang X (2011). Osteogenic effects of flavonoid aglycones from an osteoprotective fraction of Drynaria fortunei—an in vitro efficacy study. Phytomedicine.

[39] Wei Y (2011). Catechin stimulates osteogenesis by enhancing PP2A activity in human mesenchymal stem cells. Osteoporos. Int..

[40] He Y (2019). Glucagon like peptide 2 has a positive impact on osteoporosis in ovariectomized rats. Life Sci..

[41] Guruvayoorappan C, Kuttan G (2008). (+)-Catechin inhibits tumour angiogenesis and regulates the production of nitric oxide and TNF-α in LPS-stimulated macrophages. Innate Immun..

[42] Li T, Li F, Liu X, Liu J, Li D (2019). Synergistic anti-inflammatory effects of quercetin and catechin via inhibiting activation of TLR4–MyD88-mediated NF-κB and MAPK signaling pathways. Phytother. Res..

[43] Wang YG, Jiang LB, Gou B (2017). Protective effect of vanillic acid on ovariectomy-induced osteoporosis in rats. Afr. J. Tradit. Complement. Altern. Med..

[44] Tanaka T (2019). Anti-osteoporotic effects of syringic acid and vanilic acid in the extracts of waste beds after mushroom cultivation. J. Biosci. Bioeng..

[45] Mattila PT, Svanberg MJ, Mäkinen KK, Knuuttila ML (1996). Dietary xylitol, sorbitol and D-mannitol but not erythritol retard bone resorption in rats. J. Nutr..

[46] Cornish J (2008). Modulation of osteoclastogenesis by fatty acids. Endocrinology.

[47] Chauhan S (2018). In-vitro osteoblast proliferation and in-vivo anti-osteoporotic activity of Bombax ceiba with quantification of Lupeol, gallic acid and β-sitosterol by HPTLC and HPLC. BMC Complement. Altern. Med..

[48] Pereira JV, Modesto-Filho J, deFAgra, M. & Barbosa-Filho, J. M. (2002). Plant and plant-derived compounds employed in prevention of the osteoporosis. Acta Farmaceut. Bonaerense.

[49] Min J (2018). Analysis of anti-osteoporosis function of chlorogenic acid by gene microarray profiling in ovariectomy rat model. Biosci. Rep..

[50] Zhou RP (2016). Chlorogenic acid prevents osteoporosis by Shp2/PI3K/Akt pathway in ovariectomized rats. PLoS ONE.

[51] Gür A (2002). Possible pathogenetic role of new cytokines in postmenopausal osteoporosis and changes during calcitonin plus calcium therapy. Rheumatol. Int..

[52] Armour KE, Van'T Hof RJ, Grabowski PS, Reid DM, Ralston SH (1999). Evidence for a pathogenic role of nitric oxide in inflammation-induced osteoporosis. J. Bone Miner. Res..

[53] Mancini L, Moradi-Bidhendi N, Becherini L, Martineti V, MacIntyre I (2000). The biphasic effects of nitric oxide in primary rat osteoblasts are cGMP dependent. Biochem. Biophys. Res. Commun..

[54] Damoulis PD, Hauschka PV (1994). Cytokines induce nitric oxide production in mouse osteoblasts. Biochem. Biophys. Res. Commun..

[55] Van'T Hof RJ, Ralston S (2001). H. Nitric oxide and bone. Immunology.

[56] Mogi M, Kinpara K, Kondo A, Togari A (1999). Involvement of nitric oxide and biopterin in proinflammatory cytokine-induced apoptotic cell death in mouse osteoblastic cell line MC3T3-E1. Biochem. Pharmacol..

[57] Navarro VJ (2017). Liver injury from herbal and dietary supplements. Hepatology.

[58] Guañabens N, Parés A (2018). Osteoporosis in chronic liver disease. Liver Int..

[59] Scholz S (2013). Zebrafish embryos as an alternative model for screening of drug-induced organ toxicity. Arch. Toxicol..

[60] Korzh S (2008). Requirement of vasculogenesis and blood circulation in late stages of liver growth in zebrafish. BMC Dev. Biol..

[61] Nam H-S (2016). Expression of miRNA-122 induced by liver toxicants in zebrafish. BioMed Res. Int..

[62] Thiagarajan SK (2019). Evaluation of the effect of aqueous Momordica charantia Linn. extract on zebrafish embryo model through acute toxicity assay assessment. Evid.-Based Complement. Altern. Med..

[63] Karani L, Tolo F, Karanja S, Khayeka C (2013). Safety of Prunus africana and Warburgia ugandensis in asthma treatment. S. Afr. J. Bot..

[64] Gathumbi P, Mwangi J, Mugera G, Njiro S (2000). Biochemical and haematological changes mediated by a chloroform extract of Prunus africana stem bark in rats. Pharm. Biol..

