# Supplementary material for: GC–MS and LC-TOF–MS profiles, toxicity, and macrophage-dependent in vitro anti-osteoporosis activity of Prunus africana (Hook f.) Kalkman Bark
Source: Sci Rep. 2022 Apr 29;12:7044. doi: 10.1038/s41598-022-10629-7 (PMC9054796; doi:10.1038/s41598-022-10629-7)
Supplement: Supplementary file 1 — Supplementary Information. [file 41598_2022_10629_MOESM1_ESM.docx]

**Supplementary Table Y. Phytochemical components identified in the stem bark of *Prunus africana* by LC-TOF-MS analysis with positive ion mode (over 90% of library score)**

| **No.** | **Name** | **Mass**  **[M-Na]^+^** | **Founded Mass** | **Error (ppm)** | **RT (min)** | **Founded RT (min)** | **RT Delta (min)** | **Library Score (%)** | **Area** |
| --- | --- | --- | --- | --- | --- | --- | --- | --- | --- |
| 1 | Astragalin | 449.1083 | 449.1083 | 0.06 | 23.25 | 23.22 | 0.02 | 100.00 | 1129.49 |
| 2 | Chlorogenic acid | 355.1024 | 355.1024 | 0.05 | 13.16 | 13.14 | 0.02 | 100.00 | 2289.52 |
| 3 | Coproporphyrin I | 655.4932 | 655.4961 | 4.46 | 1.19 | 1.32 | 0.13 | 100.00 | 1974.83 |
| 4 | Hyperin | 465.1033 | 465.1033 | -0.11 | 23.12 | 23.12 | 0.00 | 100.00 | 3329.70 |
| 5 | Luteoloside | 449.1787 | 449.1787 | 0.02 | 22.56 | 22.56 | 0.01 | 100.00 | 4448.29 |
| 6 | Mesoporphyrin IX | 567.2814 | 567.2809 | -0.89 | 42.57 | 42.56 | 0.01 | 100.00 | 1217.89 |
| 7 | Naringenin | 273.0762 | 273.0762 | 0.17 | 33.74 | 33.73 | 0.00 | 100.00 | 6559.30 |
| 8 | Isovitexin | 433.1130 | 433.1132 | 0.40 | 22.51 | 22.51 | 0.01 | 99.82 | 1850.40 |
| 9 | 1-Methyladenosine | 282.0488 | 282.0488 | -0.29 | 2.20 | 2.21 | 0.01 | 99.72 | 1499.95 |
| 10 | Daidzin | 417.1185 | 417.1183 | -0.36 | 30.77 | 30.78 | 0.00 | 99.69 | 1458.58 |
| 11 | Apigenin-7-glucoside | 433.1128 | 433.1125 | -0.63 | 25.72 | 25.73 | 0.01 | 99.65 | 479.83 |
| 12 | 10-Gingerol +Na | 373.1834 | 373.1835 | 0.36 | 16.27 | 16.30 | 0.02 | 99.64 | 2217.38 |
| 13 | Guanosine | 284.1322 | 284.1339 | 5.87 | 6.70 | 6.93 | 0.23 | 99.56 | 307.75 |
| 14 | Catechin | 291.0867 | 291.0869 | 0.46 | 17.55 | 17.56 | 0.00 | 99.52 | 170450.66 |
| 15 | Sesamoside +Na | 443.1655 | 443.1662 | 1.52 | 3.46 | 3.46 | 0.00 | 99.41 | 11807.63 |
| 16 | Adenosine 2',3'-cyclic phosphate | 330.0601 | 330.0601 | -0.05 | 7.70 | 7.70 | 0.00 | 99.34 | 2341.71 |
| 17 | Ononin | 431.1341 | 431.1345 | 0.86 | 32.94 | 32.93 | 0.00 | 99.32 | 507.23 |
| 18 | Gingerglycolipid B +Na | 701.3726 | 701.3726 | 0.04 | 40.45 | 40.46 | 0.01 | 99.22 | 3348.02 |
| 19 | Sodium glycodeoxycholate | 472.2941 | 472.2939 | -0.38 | 43.27 | 43.26 | 0.01 | 99.11 | 3541.99 |
| 20 | Quercetin | 303.0508 | 303.0507 | -0.10 | 31.39 | 31.39 | 0.01 | 99.06 | 60207.55 |
| 21 | Rutin | 611.1604 | 611.1607 | 0.54 | 22.23 | 22.23 | 0.00 | 99.01 | 2023.50 |
| 22 | Vardenafil | 489.3213 | 489.3212 | -0.22 | 34.06 | 34.08 | 0.01 | 98.92 | 1884.40 |
| 23 | Uridine | 245.0771 | 245.0770 | -0.37 | 5.01 | 5.03 | 0.02 | 98.88 | 5220.61 |
| 24 | Ziprasidone | 413.2665 | 413.2664 | -0.34 | 44.48 | 44.48 | 0.00 | 98.71 | 22284.81 |
| 25 | Epicatechin | 291.0869 | 291.0870 | 0.41 | 15.06 | 15.06 | 0.00 | 98.44 | 59352.61 |
| 26 | Reserpine | 609.4508 | 609.4512 | 0.63 | 42.29 | 42.27 | 0.02 | 98.33 | 3452.31 |
| 27 | Glycitin | 447.1289 | 447.1290 | 0.28 | 27.03 | 27.04 | 0.01 | 98.31 | 11444.60 |
| 28 | Dihydroquercetin | 305.0661 | 305.0662 | 0.29 | 22.96 | 22.96 | 0.00 | 98.28 | 2054.38 |
| 29 | Linthospermic Acid +Na | 561.3971 | 561.3969 | -0.35 | 42.78 | 42.78 | 0.00 | 98.28 | 11983.58 |
| 30 | Sophoricoside | 433.1136 | 433.1138 | 0.57 | 24.01 | 23.66 | 0.35 | 98.23 | 554.28 |
| 31 | Metoprolol | 268.0827 | 268.0830 | 0.96 | 10.88 | 10.86 | 0.02 | 98.12 | 591.99 |
| 32 | Glycyrrhetinic acid | 471.3470 | 471.3473 | 0.65 | 41.73 | 41.72 | 0.00 | 97.88 | 7218.81 |
| 33 | Corosolic acid | 473.3510 | 473.3622 | 23.69 | 41.47 | 41.98 | 0.51 | 97.86 | 14316.52 |
| 34 | Solanesol | 631.4891 | 631.4887 | -0.67 | 1.38 | 1.38 | 0.00 | 97.85 | 6175.36 |
| 35 | Ginkgolide B | 425.3603 | 425.3603 | 0.02 | 0.65 | 0.67 | 0.02 | 97.83 | 15476.45 |
| 36 | Sorbitol | 183.1010 | 183.0863 | -80.34 | 14.46 | 15.31 | 0.85 | 97.64 | 483.97 |
| 37 | Evodiamine | 304.1446 | 304.1446 | 0.12 | 39.09 | 39.08 | 0.01 | 97.55 | 471.42 |
| 38 | Gamithromycin | 777.5825 | 777.5961 | 17.46 | 0.32 | 0.33 | 0.00 | 97.46 | 3582.65 |
| 39 | Acacetin | 285.0763 | 285.0763 | 0.07 | 27.03 | 27.04 | 0.01 | 97.42 | 14273.33 |
| 40 | Reserpine | 609.2317 | 609.2320 | 0.47 | 30.50 | 30.53 | 0.03 | 97.17 | 6509.41 |
| 41 | Pinocembrin | 257.0813 | 257.0814 | 0.40 | 38.04 | 38.03 | 0.01 | 97.11 | 830.38 |
| 42 | Pratensein-7-O-glucoside | 463.1241 | 463.1243 | 0.31 | 26.33 | 26.32 | 0.01 | 96.64 | 13758.50 |
| 43 | Procyanidin B2 | 579.1499 | 579.1498 | -0.14 | 16.55 | 16.55 | 0.00 | 96.63 | 124815.22 |
| 44 | Fluthiacet-methyl | 404.1532 | 404.1536 | 0.82 | 10.26 | 10.25 | 0.01 | 96.23 | 1531.64 |
| 45 | 5alpha-Cholestanone | 387.1625 | 387.1627 | 0.53 | 13.81 | 13.80 | 0.01 | 96.10 | 3708.21 |
| 46 | Diniconazole | 326.2692 | 326.2691 | -0.38 | 42.52 | 42.53 | 0.01 | 95.54 | 9384.80 |
| 47 | Ophiopogonin D | 855.5083 | 855.5079 | -0.45 | 42.48 | 42.47 | 0.00 | 95.47 | 2175.68 |
| 48 | Emodin-8-glucoside +Na | 455.2252 | 455.2259 | 1.47 | 27.31 | 27.33 | 0.02 | 94.96 | 1054.25 |
| 49 | Betulonic acid | 455.1315 | 455.1314 | -0.06 | 34.39 | 34.37 | 0.02 | 93.95 | 1111.10 |
| 50 | Tuberostemonine | 376.3059 | 376.3057 | -0.51 | 34.51 | 34.50 | 0.01 | 93.93 | 580.29 |
| 51 | Arenobufagin | 417.1981 | 417.1984 | 0.80 | 2.27 | 2.29 | 0.02 | 93.32 | 1206.81 |
| 52 | Fenhexamid | 302.1290 | 302.1292 | 0.67 | 18.27 | 18.28 | 0.01 | 93.15 | 7730.15 |
| 53 | (+)-Tetrandrine | 623.2464 | 623.2462 | -0.34 | 33.88 | 33.93 | 0.05 | 92.60 | 498.62 |
| 54 | Galangin | 271.0965 | 271.0965 | 0.17 | 18.94 | 18.95 | 0.01 | 92.47 | 2630.10 |
| 55 | Sclareol +Na | 331.0452 | 331.0456 | 1.24 | 35.32 | 35.32 | 0.00 | 91.99 | 342.09 |
| 56 | Oxadixyl | 279.0936 | 279.0938 | 0.82 | 42.50 | 42.21 | 0.29 | 91.68 | 3346.41 |
| 57 | Donepezil | 380.3521 | 380.3524 | 0.81 | 44.23 | 44.24 | 0.02 | 91.60 | 761.53 |
| 58 | Lasalocid A | 613.4832 | 613.4832 | -0.10 | 44.69 | 44.71 | 0.02 | 91.45 | 12507.45 |
| 59 | Thiophanate | 371.1497 | 371.1498 | 0.13 | 38.97 | 38.99 | 0.02 | 91.32 | 1461.80 |
| 60 | Norbuprenorphine | 414.1455 | 414.1454 | -0.43 | 8.20 | 8.21 | 0.02 | 90.90 | 1286.71 |
| 61 | Bisdemethoxycurcumin | 309.0972 | 309.0972 | -0.18 | 21.70 | 21.70 | 0.00 | 90.82 | 35682.67 |
| 62 | Resibufogenin | 385.1638 | 385.1636 | -0.37 | 26.54 | 26.53 | 0.01 | 90.54 | 1339.03 |
| 63 | Cepharanthine | 607.2148 | 607.2149 | 0.06 | 32.75 | 32.76 | 0.01 | 90.44 | 5405.98 |
| 64 | Hydroxygenkwanin | 301.0348 | 301.0347 | -0.06 | 33.08 | 33.08 | 0.00 | 90.30 | 1716.19 |
| 65 | Senecionin | 336.1057 | 336.1057 | 0.26 | 9.51 | 9.42 | 0.09 | 90.18 | 56887.10 |

**Supplementary Table Z. Phytochemical components identified in the stem bark of *Prunus africana* by LC-TOF-MS analysis with negative ion mode (over 90% of library score)**

| **No.** | **Name** | **Mass**  **[M-H]^−^** | **Founded Mass** | **Error**  **(ppm)** | **RT**  **(min)** | **Founded RT (min)** | **RT Delta (min)** | **Library Score (%)** | **Area Area** |
| --- | --- | --- | --- | --- | --- | --- | --- | --- | --- |
| 1 | Pedunculoside +HCOOH | 695.3693 | 695.3696 | 0.47 | 34.39 | 34.38 | 0.00 | 100.00 | 20498.21 |
| 2 | Luteoloside | 447.0696 | 447.0695 | -0.27 | 23.18 | 23.21 | 0.03 | 100.00 | 1513.48 |
| 3 | Hexadecanedioic acid | 285.1910 | 285.1909 | -0.10 | 40.49 | 40.48 | 0.01 | 100.00 | 4854.99 |
| 4 | Guanosine | 282.0683 | 282.0684 | 0.10 | 6.66 | 6.65 | 0.01 | 100.00 | 7969.05 |
| 5 | Betulonicacid | 453.2775 | 453.2776 | 0.07 | 42.97 | 42.97 | 0.00 | 100.00 | 1209.40 |
| 6 | Lithocholic acid | 375.2538 | 375.2543 | 1.17 | 32.40 | 32.40 | 0.00 | 99.96 | 786.47 |
| 7 | Amber Acid | 117.0113 | 117.0112 | -0.26 | 5.23 | 5.24 | 0.01 | 99.92 | 12654.45 |
| 8 | Hypericin | 503.3126 | 503.3128 | 0.27 | 35.64 | 35.65 | 0.01 | 99.80 | 54689.29 |
| 9 | Maleic acid | 114.9957 | 114.9957 | 0.14 | 4.14 | 4.14 | 0.00 | 99.75 | 13276.86 |
| 10 | Ononin +HCOOH | 475.1936 | 475.1946 | 2.08 | 23.22 | 23.23 | 0.00 | 99.57 | 1465.31 |
| 11 | Polygalaxanthone IX | 551.1494 | 551.1494 | 0.11 | 31.87 | 31.90 | 0.02 | 99.56 | 2257.19 |
| 12 | Isorhamnetin | 315.1643 | 315.1721 | 24.87 | 39.17 | 39.01 | 0.16 | 99.49 | 231.95 |
| 13 | Salvianolic acid B | 717.3272 | 717.3292 | 2.79 | 26.81 | 26.79 | 0.02 | 99.37 | 778.22 |
| 14 | Procyanidin B2 | 577.1062 | 577.1063 | 0.16 | 22.14 | 22.14 | 0.00 | 99.26 | 43817.03 |
| 15 | Chenodeoxycholic acid | 391.0822 | 391.0835 | 3.28 | 3.58 | 3.59 | 0.01 | 99.25 | 6157.16 |
| 16 | Vitexin | 431.1690 | 431.1692 | 0.56 | 22.44 | 22.43 | 0.01 | 99.20 | 4598.51 |
| 17 | Polyphyllin VI | 737.1994 | 737.1995 | 0.09 | 6.11 | 6.13 | 0.02 | 99.08 | 5400.46 |
| 18 | Catechin | 289.0553 | 289.0553 | 0.32 | 17.52 | 17.49 | 0.03 | 99.08 | 161539.21 |
| 19 | Cyasterone | 519.3070 | 519.3069 | -0.13 | 37.71 | 37.71 | 0.01 | 99.06 | 47534.32 |
| 20 | Apigenin-7-glucoside | 431.2055 | 431.2057 | 0.40 | 25.58 | 25.56 | 0.02 | 99.05 | 486.69 |
| 21 | Glycyrrhetinic acid | 469.2723 | 469.2725 | 0.53 | 41.17 | 41.17 | 0.00 | 99.02 | 2756.51 |
| 22 | Daidzin | 415.2118 | 415.2119 | 0.27 | 34.15 | 34.17 | 0.01 | 98.96 | 1328.85 |
| 23 | Cryptochlorogenic acid | 353.0684 | 353.0682 | -0.56 | 16.69 | 16.70 | 0.01 | 98.88 | 88290.11 |
| 24 | Roburic acid | 439.0440 | 439.0439 | -0.31 | 28.92 | 28.93 | 0.01 | 98.87 | 2430.87 |
| 25 | Phloridzin | 435.1063 | 435.1063 | -0.12 | 26.27 | 26.29 | 0.02 | 98.68 | 6703.49 |
| 26 | Calceorioside B | 477.1001 | 477.1003 | 0.30 | 11.75 | 11.75 | 0.00 | 98.57 | 1208.20 |
| 27 | Glycetein | 283.0450 | 283.0450 | -0.30 | 27.41 | 27.41 | 0.00 | 98.41 | 605.42 |
| 28 | Amygdalin | 502.1302 | 502.1302 | -0.02 | 15.21 | 15.24 | 0.02 | 98.32 | 157251.89 |
| 29 | Gingerglycolipid B +HCOOH | 723.3491 | 723.3491 | 0.11 | 40.49 | 40.49 | 0.00 | 98.21 | 9221.87 |
| 30 | Ziyuglycoside II | 603.1448 | 603.1464 | 2.64 | 3.23 | 4.14 | 0.91 | 98.14 | 806.92 |
| 31 | Estriol | 287.2065 | 287.2065 | 0.18 | 39.37 | 39.38 | 0.00 | 98.12 | 1438.03 |
| 32 | Phenobarbital | 230.9725 | 230.9729 | 1.74 | 41.80 | 41.00 | 0.80 | 98.06 | 350.68 |
| 33 | Heterophyllin B | 777.1952 | 777.1933 | -2.54 | 8.39 | 8.37 | 0.01 | 98.02 | 4496.57 |
| 34 | Saikosaponin D | 779.2574 | 779.2575 | 0.10 | 32.89 | 32.88 | 0.01 | 98.01 | 1381.08 |
| 35 | Asiatic acid | 487.2305 | 487.2302 | -0.62 | 35.41 | 35.39 | 0.02 | 97.58 | 870.94 |
| 36 | Polygalaxanthone | 597.1541 | 597.1533 | -1.25 | 30.86 | 30.86 | 0.00 | 97.49 | 1289.62 |
| 37 | Calycosin-7-o-glucoside +HCOOH | 491.0945 | 491.0944 | -0.24 | 33.10 | 33.09 | 0.01 | 97.42 | 3412.20 |
| 38 | Phytolaccagenin | 531.2560 | 531.2561 | 0.18 | 32.49 | 32.49 | 0.00 | 97.28 | 9292.99 |
| 39 | Genistein | 269.1604 | 269.1610 | 2.06 | 34.94 | 34.90 | 0.04 | 97.02 | 382.40 |
| 40 | Phytolaccagenin +HCOOH | 577.2563 | 577.2575 | 2.09 | 23.57 | 23.57 | 0.01 | 97.00 | 1314.82 |
| 41 | Eriodictyol | 287.0397 | 287.0397 | -0.15 | 29.96 | 29.94 | 0.02 | 96.79 | 1864.55 |
| 42 | Uridine 5'-monophosphate | 323.0105 | 323.0105 | -0.13 | 8.13 | 8.13 | 0.00 | 96.39 | 23805.36 |
| 43 | Rutin | 609.1158 | 609.1154 | -0.62 | 22.23 | 22.22 | 0.01 | 96.33 | 6917.16 |
| 44 | Phytolaccagenin +HCOOH | 577.2583 | 577.2579 | -0.67 | 33.81 | 33.80 | 0.01 | 95.72 | 8528.47 |
| 45 | Apigenin | 269.0300 | 269.0301 | 0.13 | 34.11 | 34.12 | 0.01 | 95.54 | 861.34 |
| 46 | Naringenin | 271.0457 | 271.0458 | 0.64 | 24.84 | 24.82 | 0.02 | 95.37 | 3675.96 |
| 47 | Geniposide + HCOOH | 433.1848 | 433.1848 | -0.04 | 18.97 | 18.97 | 0.00 | 95.31 | 17878.59 |
| 48 | Daidzin +HCOOH | 461.0855 | 461.0860 | 0.95 | 30.75 | 30.73 | 0.01 | 94.97 | 818.85 |
| 49 | Levulinic acid | 115.0319 | 115.0320 | 0.84 | 7.26 | 7.27 | 0.01 | 94.86 | 827.93 |
| 50 | Uridine diphosphate glucose | 565.0202 | 565.0211 | 1.74 | 32.82 | 32.79 | 0.03 | 94.85 | 289.27 |
| 51 | 2-Phenylbutyric acid | 163.0303 | 163.0303 | 0.36 | 13.14 | 13.17 | 0.03 | 94.64 | 646.26 |
| 52 | Pachymic acid | 527.1422 | 527.1415 | -1.27 | 26.57 | 26.56 | 0.01 | 93.49 | 3845.48 |
| 53 | Diosmetin | 299.0030 | 299.0030 | -0.10 | 33.01 | 33.04 | 0.03 | 93.29 | 8932.48 |
| 54 | Trifolirhizin +HCOOH | 491.0943 | 491.0946 | 0.65 | 27.41 | 27.40 | 0.01 | 93.27 | 1545.12 |
| 55 | Sennoside A | 861.1291 | 861.1288 | -0.28 | 24.84 | 24.79 | 0.05 | 93.22 | 2183.58 |
| 56 | Hydroxysafflor Yellow A | 611.2046 | 611.2047 | 0.16 | 27.90 | 27.93 | 0.02 | 93.10 | 4472.09 |
| 57 | Engeletin | 433.1774 | 433.1775 | 0.30 | 29.38 | 29.38 | 0.00 | 92.95 | 2054.52 |
| 58 | Biotin | 243.0158 | 243.0160 | 0.56 | 25.72 | 25.74 | 0.02 | 92.94 | 866.08 |
| 59 | Senegenin | 535.1553 | 535.1549 | -0.74 | 27.95 | 27.96 | 0.01 | 92.63 | 434.03 |
| 60 | 4-Aminohippuric acid | 193.0022 | 193.0019 | -1.55 | 21.24 | 21.24 | 0.00 | 92.63 | 950.68 |
| 61 | Senegenin | 535.3008 | 535.3008 | 0.03 | 27.97 | 27.97 | 0.00 | 92.63 | 4634.17 |
| 62 | Epibrassinolide +HCOOH | 525.2724 | 525.2724 | -0.06 | 35.01 | 35.03 | 0.02 | 92.44 | 3303.37 |
| 63 | Picroside II | 511.2290 | 511.2288 | -0.38 | 32.19 | 32.20 | 0.01 | 92.22 | 25649.42 |
| 64 | Closantel | 661.2086 | 661.2182 | 14.49 | 29.96 | 30.57 | 0.60 | 92.12 | 1382.93 |
| 65 | Protopanaxadiol +HCOOH | 505.1816 | 505.1812 | -0.74 | 23.13 | 23.15 | 0.02 | 91.86 | 1293.96 |
| 66 | Glycocholic acid | 464.2365 | 464.2338 | -5.69 | 19.92 | 19.66 | 0.26 | 91.80 | 1416.16 |
| 67 | Emodin-8-glucoside | 431.1612 | 431.1611 | -0.27 | 31.46 | 31.45 | 0.01 | 91.41 | 2935.34 |
| 68 | Demethylzeylasteral | 479.1882 | 479.1884 | 0.38 | 22.41 | 22.41 | 0.01 | 91.32 | 1785.29 |
| 69 | Glycyrrhizic acid | 821.2661 | 821.2659 | -0.15 | 35.71 | 35.69 | 0.02 | 90.97 | 276.98 |
| 70 | Stachyose | 665.3652 | 665.3658 | 0.92 | 42.25 | 41.90 | 0.35 | 90.92 | 2196.77 |
| 71 | Kaempferol | 285.0239 | 285.0242 | 1.20 | 27.11 | 27.09 | 0.02 | 90.29 | 701.72 |
| 72 | Syringin +HCOOH | 417.1190 | 417.1187 | -0.78 | 20.94 | 20.92 | 0.01 | 90.03 | 5199.52 |
